# Advance in Drug Delivery for Ageing Skeletal Muscle

**DOI:** 10.3389/fphar.2020.01016

**Published:** 2020-07-08

**Authors:** Yi Li, Ming Chen, Yanpeng Zhao, Ming Li, Yong Qin, Shi Cheng, Yanyu Yang, Pengbin Yin, Licheng Zhang, Peifu Tang

**Affiliations:** ^1^ Department of Orthopedics, General Hospital of Chinese PLA, Beijing, China; ^2^ National Clinical Research Center for Orthopedics, Sports Medicine & Rehabilitation, Beijing, China; ^3^ The Department of Orthopedic Surgery, Second Affiliated Hospital of Harbin Medical University, Harbin, China; ^4^ College of Materials Science and Engineering, Zhengzhou University, Zhengzhou, China

**Keywords:** ageing skeletal muscle, drug delivery, extracellular vesicle, sarcopenia, skeletal muscle regeneration

## Abstract

The age-related loss of skeletal muscle, sarcopenia, is characterized by progressive loss of muscle mass, reduction in muscle strength, and dysfunction of physical performance. It has become a global health problem leading to several adverse outcomes in the ageing population. Research on skeletal muscle loss prevention and treatment is developing quickly. However, the current clinical approaches to sarcopenia are limited. Recently, novel drug delivery systems offer new possibilities for treating aged muscle loss. Herein, we briefly recapitulate the potential therapeutic targets of aged skeletal muscle and provide a concise advance in the drug delivery systems, mainly focus on the use of nano-carriers. Furthermore, we elaborately discuss the prospect of aged skeletal muscle treatment by nanotechnology approaches.

## Introduction

Skeletal muscles, as one of the largest organs in the body, are characterized by their mechanical activity required for posture, movement, and breathing (Fan et al., 2013; Pedersen, 2013). However, with the increasing age, skeletal muscle mass gradually declines and gradually loses function, leading to adverse outcomes in older people, such as high risk of falls and fractures, and damaged physical ability (Wilkinson et al., 2018; Cuesta-Triana et al., 2019). Current clinical approaches to age-related muscle loss are limited. At present, there are many studies on skeletal muscle regeneration which focus on muscle injuries, but research on strategies for age-related muscle loss is still relatively basic.

Conventionally, the research of skeletal muscle injuries mainly focuses on tissue engineering methods and cell therapies. These methods are suitable for the injury with obvious muscle defects (McKeon-Fischer et al., 2015). However, these methods are not suitable for age-related muscle loss, a kind of systemic skeletal muscle disease without local defect. In recent years, novel nano-carriers delivery systems offer new possibilities for treating systemic diseases. Even further, Rong, S et al. proposed a prospect that extracellular vesicles (EVs), including exosomes, may also be ideal drug carriers which could regulate muscle regeneration and protein synthesis. EV-based delivery systems may be potential strategies of age-related muscle loss in the future (Rong et al., 2020).

Therefore, this review will discuss the potential therapeutic targets of ageing skeletal muscle and provide a concise advance in the drug delivery systems, mainly focus on the use of nano-carriers.

## Molecular Mechansim of Ageing Skeletal Muscle Regeneration

### The Process of Skeletal Muscle Regeneration

**Figure 1:**
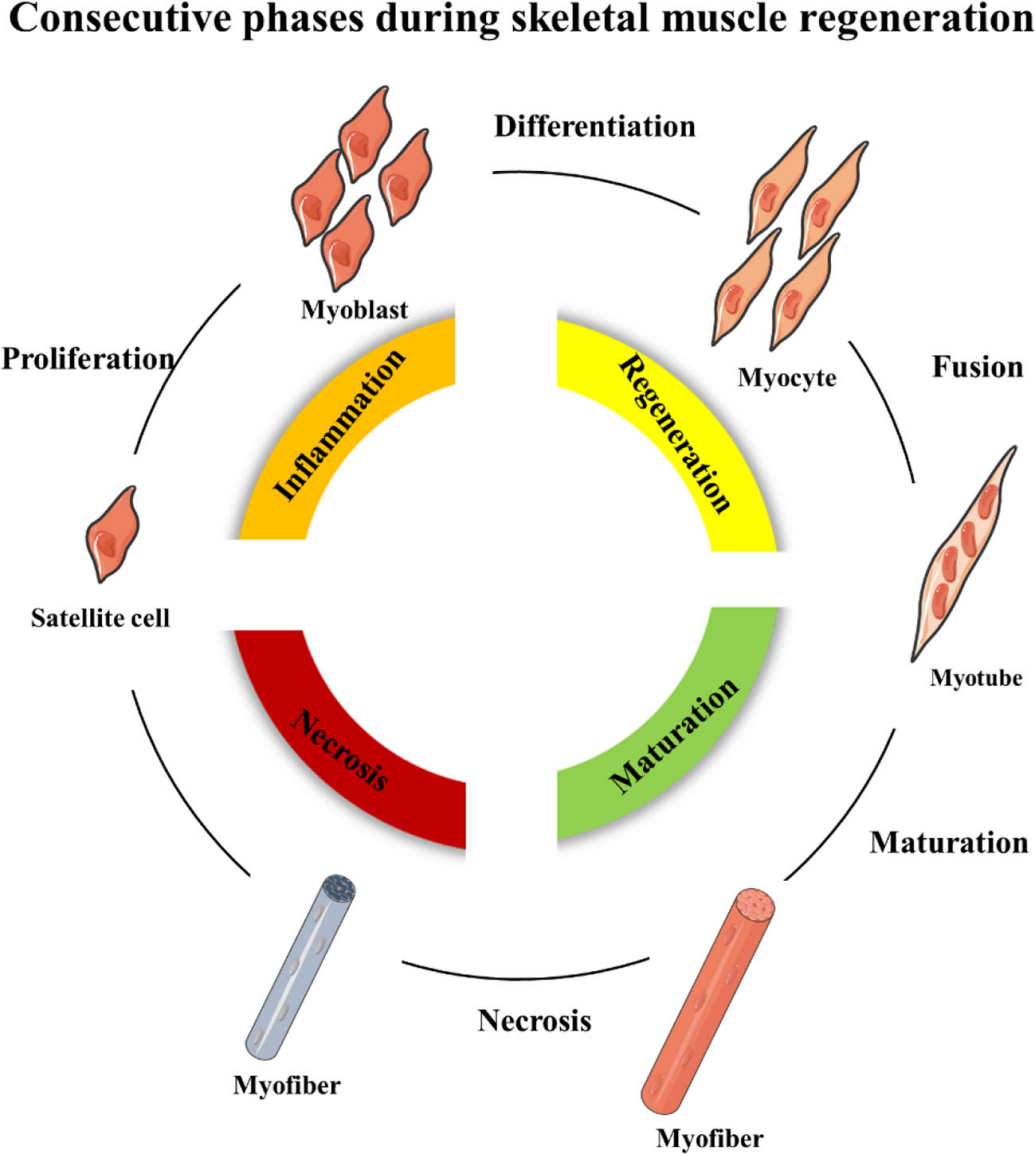
Schematic representation of the four consecutive phases during skeletal muscle regeneration.

**Figure 2:**
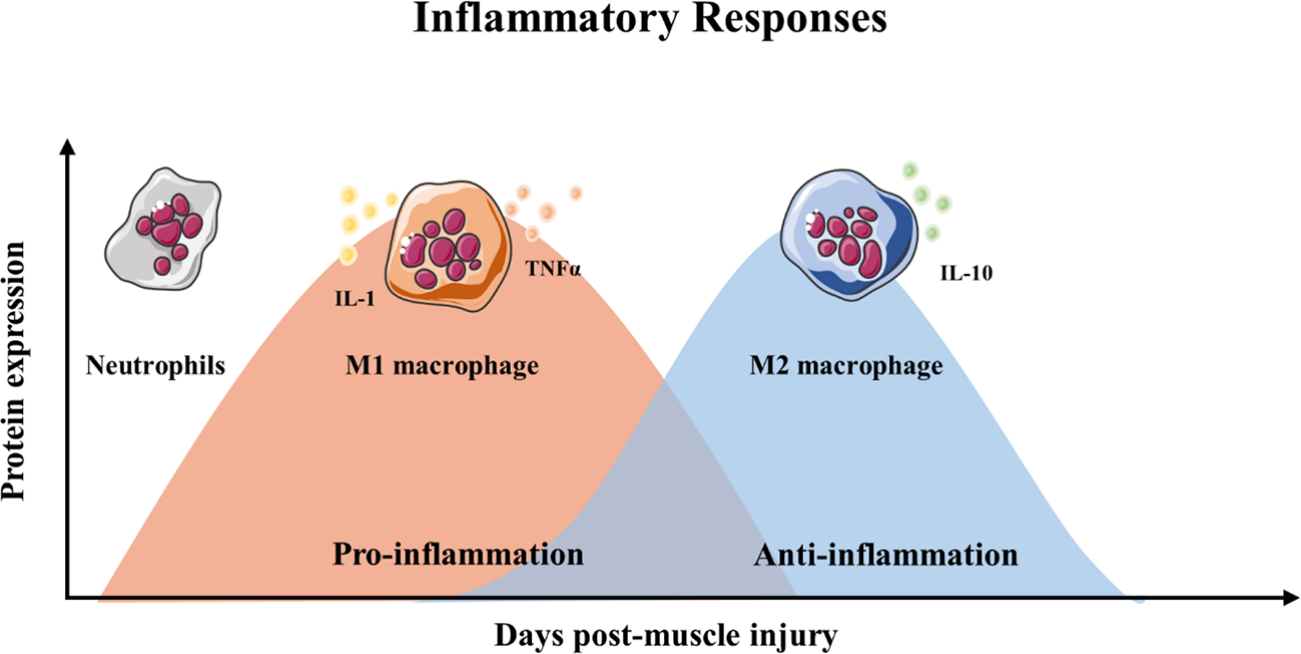
Timeline of inflammatory responses and immune cell during muscle regeneration.

Skeletal muscle regeneration includes four consecutive phases: the degeneration phase, the inflammatory phase, the phase of satellite cell differentiation, and the phase of maturation and remodeling (Barberi et al., 2013; Yin et al., 2013; Schmidt et al., 2019) **(** **Figure 1** **)**. After injury, myofibers are rapid necrosis (phase of degeneration) which induce the inflammatory phase **(** **Figure 2** **)**. The first inflammatory cells, neutrophils, are recruited to the damaged skeletal muscle within 6 h. Soon afterwards, macrophages begin to infiltrate the damaged skeletal muscle. Similar to the inflammation in other post injury parts, the infiltrating macrophages include M1 macrophages (CD68^+^/CD163^−^) and M2 macrophages (CD68^−^/CD163^+^). In the first 24 h, the M1 macrophages secrete pro-inflammatory cytokines such as TNFα and IL-1. Then, the M2 macrophages secrete anti-inflammatory cytokines such as IL-10 to attenuate the inflammatory response and promote satellite cells (SC) proliferation and differentiation. (Saini et al., 2016). In the third phase, unactivated satellite cells migrate to the target location and begin to differentiate into rapidly proliferating cells, myogenic progenitor cells (Souza et al., 2018). In this phase, several muscle-related proteins are detected with highly expression such as MyoD, Desmin, and Myf5 (Zammit, 2017; Schiaffino et al., 2018). In the maturation phase, myogenic progenitor cells differentiate into myocytes and newly myofibers occur. Meanwhile, devMHC (developmental myosin heavy chain), are detected with highly expression in this phase (Sutcu and Ricchetti, 2018). At the end of the maturation phase, the nuclei move towards the edge of newly myofibers which simultaneously increase in size (Ruparelia et al., 2019). However, when ageing occurs, the process of skeletal muscle regeneration is hindered. The main obstacles include changes in the satellite cells themselves and changes in the muscle regeneration microenvironment.

### Changes in the Ageing Satellite Cells

Satellite cells, also called muscle stem cells, are necessary for muscle regeneration and support the repair and remodeling of muscle fibers and help maintain healthy muscle mass throughout life. With ageing, satellite cells become losing self-renewal and regenerative capacity. The number of muscle stem cells expressing high level of transcription factor Pax7 (Pax7Hi) decreased during ageing (Li H. et al., 2019). Recent studies have explored changes in satellite cells from the genetic and molecular levels. Epigenetic stress response in satellite cells would limit muscle regeneration by Hoxa9 developmental signaling. After analyzing the whole genome of satellite cells from healthy individuals of different ages, somatic mutagenesis was identified as another age-related negative regulator (Schwörer et al., 2016; Franco et al., 2018). Several signaling pathways were found become aberrant during muscle ageing including RTK signaling, FGF signaling and p38 MAPK (Patel et al., 2019; Bae et al., 2020). Cdon-deficit satellite cells had impaired proliferation and lower regenerative capacity *via* aberrant integrin and FGFR signaling. In ageing satellite cells, the p38 α/β-MAPK signaling can activate high expression of p16^Ink4a^ which could inhibit satellite cells self-renew (Almada and Wagers, 2016; Zhu et al., 2019). Emerging studies found that ageing leads to the asymmetric division of satellite cells (Garcia-Prat and Munoz-Canoves, 2017; Feige et al., 2018; Hwang and Brack, 2018). Garcia-Prat et al. found that autophagic activity is also impaired in aged satellite cells, resulting in the accumulation of impaired proteins and mitochondria, leading to the dysfunction and reduced number of satellite cells (García-Prat et al., 2016). Oxidative stressors, especially reactive oxygen species (ROS), have been thought to have a negative effect on skeletal muscle (Baraibar et al., 2013). The oxidative capacity of skeletal muscles is closely related to general physical performance. Overexpressed ROS cause oxidative stress and mitochondrial dysfunction in cells, which eventually induce apoptosis (Zhou et al., 2018; Szentesi et al., 2019).

### Changes in the Aged Muscle Microenvironment

During the ageing process, the microenvironment of muscle tissue changes, which affects the process of self-repair. Immune regulation is closely related to muscle regeneration. The numbers of myeloid lineage cells in their skeletal muscles increased during ageing, including macrophages and granulocytes (Li et al., 2020). When immune cells and factors change in the muscle microenvironment, muscle regeneration will be affected (Livshits and Kalinkovich, 2019). Immune senescence includes a systemic, chronic, low-grade pro-inflammatory state which is also known as inflammageing (Wilson et al., 2017). The proportion of serum inflammatory cytokines begins to change. The proportion of pro-inflammatory cytokines, such as IL-1β, IL-6 and TNFα, are gradually increasing. In contrast, the proportion of anti-inflammatory cytokines are gradually decreased (Rezus et al., 2020). The reasons for inflammageing are complex, including changes in monocyte and lymphocyte phenotypes. Meanwhile, obesity is also involved in the process of immuneageing, in which the expression of pro-inflammatory factors such as leptin increases and the expression of anti-inflammatory factors such as adiponectin decreases (Wilson et al., 2017; Weyh et al., 2020). Lukjanenko L et al. found that fibro-adipogenic progenitors indirectly affect the functions of satellite cells during ageing. WNT1 Inducible Signaling Pathway Protein 1 (WISP1) secreted by fibro-adipogenic progenitors was a key matricellular communicator to repair regenerative capacity (Lukjanenko et al., 2019).

## Potential Therapeutic Targets of Ageing Skeletal Muscle

Currently, the only validated treatment of aged skeletal muscle is exercise, which can reverse different types of muscle ageing to some extent. However, for patients undergoing long-term bed rest or with other clinical complications, they are generally not recommended to exercise. Therefore, there is a need to develop new treatments for old patients to reduce skeletal muscle loss and restore muscle function. (Cohen et al., 2015). Many studies have identified various biological pathways and targets that may promote skeletal muscle regeneration, and some drugs have been tested in clinical trials. For example, A phase 2 randomized study involved 170 aged women diagnosed with sarcopenia and moderate physical dysfunction showed a significant increase in lean body mass but fail to improve muscle strength or function after receiving MK-0773, the selective androgen receptor modulator (SARM) (Papanicolaou et al., 2013). Another cohort involved in 400 individuals aged ≥50 years treated with total hip arthroplasty for osteoarthritis. After receiving LY2495655, a humanized monoclonal antibody targeting myostatin, no meaningful difference in muscle strength, physical performance was found compared to placebo (Woodhouse et al., 2016).To date, results from trials have shown less success for improving muscle strength or patient physical function (Rooks and Roubenoff, 2019). As research continues, new targets have emerged in recent years, giving prospect for disease treatment. Here, we summarize the latest promising targets for treating muscle ageing.

### Anti-Myostatin

Myostatin (MSTN), functioning as an endogenous regulator for the skeletal muscle growth, has drawn wide attention as a novel target. Myostatin can combine with activin receptors type IIB (ActRIIB) on the myofibers membrane which regulate skeletal muscle mass and function (White and LeBrasseur, 2014). Myostatin may also directly affect skeletal muscle mass by suppressing muscle regeneration (Ge et al., 2020). Because myostatin significantly regulates muscle regeneration, it has become a promising target for preventing the decline and dysfunction of skeletal muscle function for many years (Polkey et al., 2019). Several clinical trials have shown positive results through direct inhibiting the expression of myostatin. LY2495655, a humanized MSTN antibody, could increase appendicular lean mass in a randomised, phase 2 clinical trial (Becker et al., 2015). Bimagrumab is another human monoclonal antibody which targets activin type II receptors (ActRII). A 24-week, randomized, double-blind study, which involved 40 individuals, showed that treat with bimagrumab over 16 weeks could increase muscle mass and strength in older adults with sarcopenia. However, a multicentre, double-blind, placebo-controlled study, which involved 251 individuals, evaluated the safety and efficacy of intravenous bimagrumab in inclusion body myositis. The results indicated the treatment of bimagrumab did not meet the primary endpoint as no significant difference between bimagrumab and placebo in 6-min walking distance (6MWD) (Furrer and Handschin, 2019; Hanna et al., 2019; Hardee and Lynch, 2019). The above clinical research shows that large-scale multi-center clinical research is still needed to verify the efficacy and adaptive diseases of anti-myostatin treatment.

### Anti-Inflammatory Cytokine

Since the immune cells are deeply involved in skeletal reconstruction process, the immune cells and inflammatory factors may become the target of improving aged skeletal muscle. Huang, S et al. observed the highly expression of TNF-α and decreased expression of IL-10 in soleus muscle after treating with doxorubicin, an anti-tumor drug which could cause skeletal muscle loss in cancer patients. More importantly, doxorubicin may cause the absent of M1 macrophage during the inflammatory phase (Huang S. et al., 2017). Lee, C. et al. found Magnolol, a kind of anti-cancer drug, could attenuate the imbalance of M1/M2c macrophages to inhibit muscle loss (Lee et al., 2020). Further, Liao, Z.H., et al. found that regulating the functions of Treg cells would also be an important measure to promote muscle regeneration. They treated cardiotoxin (CTX)-induced muscle injury by estrogen and the results indicated estrogen could suppress immune response and reverse phenotypes of monocytes/macrophages by regulating the function of Treg cells, and suppressing Th1 response in the inflammatory phase (Liao et al., 2019). Administration of exogenous anti-inflammatory factors may also improve skeletal muscle regeneration. Costamagna, D et al. found the IL-4 treatment would improve the performances and prolonged survival of colon carcinoma-bearing (C26) mice. IL-4 could rescue muscle mass by increasing protein synthesis and promote myogenesis (Costamagna et al., 2020). Du H et al. investigated that overexpression of ADAMTS1 in macrophages could active satellite cell and promote muscle regeneration by reduces Notch signaling (Du et al., 2017). Although a large number of studies have suggested that immune cells and immune factors play an important role in promoting muscle regeneration, the role of the immune system in ageing muscle loss is unknown. Therefore, further research is needed to further explore the immune microenvironment around ageing skeletal muscles for possible therapeutic targets.

### Nucleic Acids

Nucleic acids, particularly non-encoding nucleic acid, act as a key modulator of skeletal muscle proliferation and regeneration. It is noteworthy that a group of muscle- specific miRNAs, myomiRNAs, are closely related to skeletal muscle development and regeneration, including miR-133, miR-206, miR-208b, and miR-499. Iannone, F et al. investigated that the under-expression of miR-133b may contribute to the impaired regenerative capacity of satellite cells (Iannone et al., 2020). The expression of miR-206 increased during muscle regeneration result in the inhibition of Pax3, Pax7, and c-Met. These downstream genes subsequently affect myogenic differentiation (McCarthy, 2008).

As EVs are closely related to exercise, researchers have confirmed that different types of exercise would release a large number of EVs into the blood circulation. Since the discovery of EVs can transfer functional non-encoding nucleic acid, EV-associated nucleic acid may play a key role in muscle cell-cell communication (Yin et al., 2018). Fulzele, S. et al. found a significant increase in expression of senescence-associated EVs-derived miR-34a in skeletal muscle from ageing mice. The results indicated that senescence‐associated EVs secreted from skeletal muscle may directly reduce stem cell populations *via* their microRNA cargo (Fulzele et al., 2019). As the growing understanding of EVs, more therapeutic targets may be constantly discovered.

### Bone-Derived Factors

**Figure 3:**
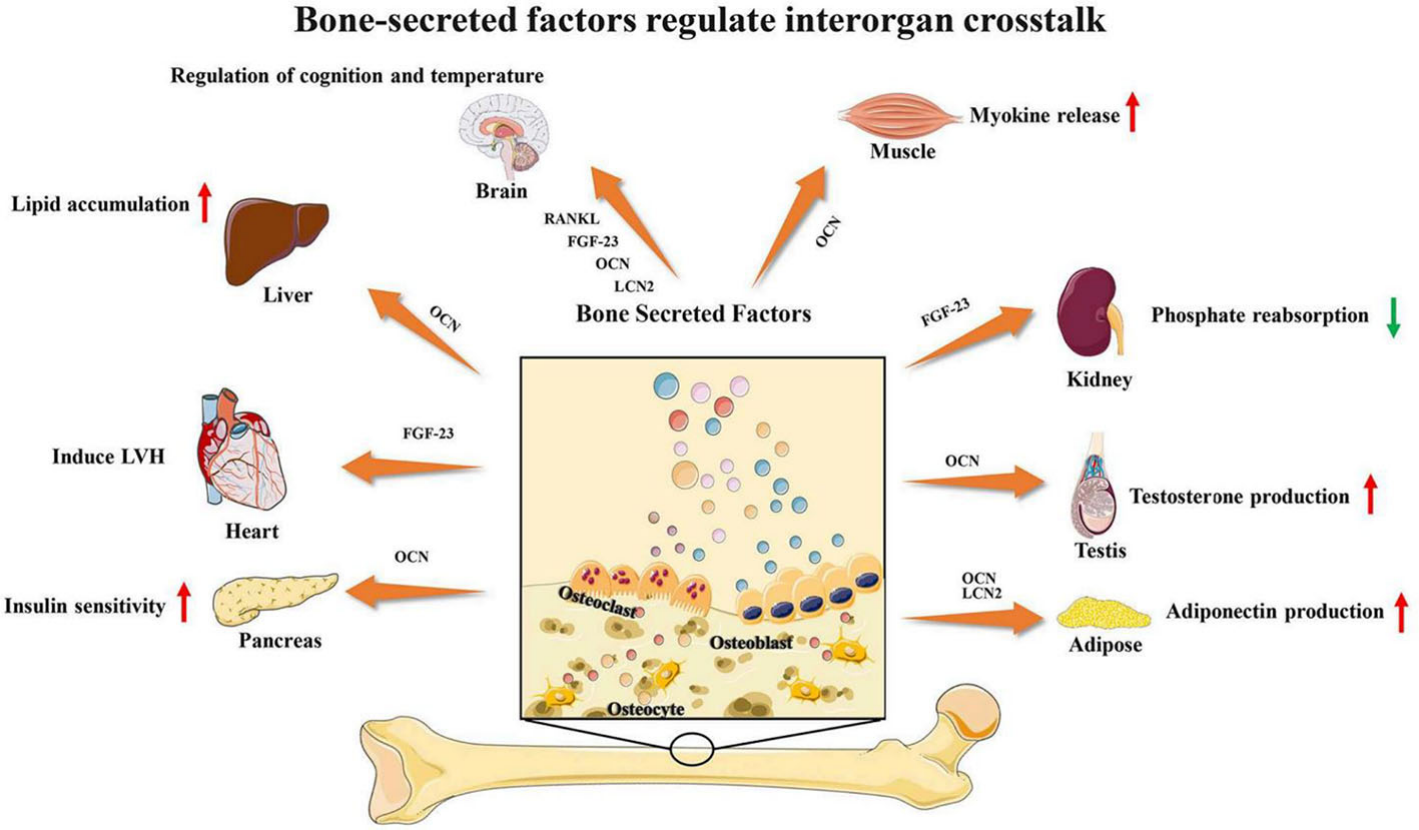
Bone-secreted factors regulate interorgan crosstalk. Reproduced, with permission, from (Li Y. et al., 2019).

Bone and muscle are closely related, and many studies have found that bone and muscle interact on each other. As emerging evidence supports the concept that bone would act as an endocrine organ, bone-secreted factors may be an important target for muscle regeneration **(** **Figure 3** **)**. Osteocalcin (OCN) is currently the most widely studied bone-derived factor. OCN regulates the uptake and catabolism of nutrients in muscle during exercise by promoting the release of interleukin-6 from myofibers. More importantly, the muscle mass increased significantly after giving exogenous OCN in older mice. These findings strongly suggest that OCN would be a promising therapeutic target for ageing muscle regeneration(Mera et al., 2016a; Mera et al., 2016b). In contrast, transforming growth factor (TGF)-β derived from bone surface was a negative regulator which contributes to muscle weakness (Waning et al., 2015). Sclerostin is mainly secreted by osteocytes and osteoblasts which is a key inhibitor of Wnt signaling. Sclerostin could inhibit myogenic differentiation by activating Wnt/β-catenin pathway *in vitro* experiment. Hesse E et al. found that sclerostin antibody would alleviate muscle weakness and improve skeletal muscle function (Huang J. et al., 2017; Hesse et al., 2019). The receptor-activator of nuclear factor κB/receptor-activator of nuclear factor κB ligand/osteoprotegerin (RANK/RANKL/OPG) is a key pathway for bone remodeling. Recent studies found that bone derived RANKL inhibitor could significantly improve skeletal muscle function and restore bone mass. OPG-deficient mice displayed reduced muscle weakness with selective atrophy of fast-twitch-type IIb myofibers. After using RANKL inhibitors, represented by Denosumab, patients have improved handgrip strength which indicate RANKL inhibitors may be an important method for treating muscle diseases (Dufresne et al., 2018; Bonnet et al., 2019; Hamoudi et al., 2020).

Although there are many therapeutic targets for ageing skeletal muscle, a very important point is how to accurately deliver functional nucleic acids or proteins. Obviously, the current clinical trials for muscle regeneration have encountered some difficulties. For example, the anti-myostatin drug has not achieved the expected effect. The results of clinical trials suggest that the exploration of functional molecules may not achieve good therapeutic effects. How to accurately deliver and release functional molecules may be another important issue to be explored. We believe that building an appropriate delivery system for muscle regeneration is another area worthy of in-depth study.

## Drug Delivery System for Ageing Skeletal Muscle Regeneration

To treat aged skeletal muscle dysfunction, appropriate drug delivery system that loads with potential therapeutic factors would be highly desired. Here, we summarize the current promising drug delivery systems, and future studies may load different active factors on the basis of these systems.

### Adeno-Associated Virus (AAV)

Adeno-associated viral (AAV) vectors are currently developing rapidly in the treatment of gene-related muscle disorders. Natural or engineered viral capsids have been successfully constructed by transferring nucleic acids, antibodies or gene-editing machinery *via* various routes of administration successfully (Lisowski et al., 2015; Naso et al., 2017). Currently, AAV appears to be the most promising vector for gene therapy, especially the treatment of Duchenne muscular dystrophy and Spinal muscular atrophy (SMA) (Groen et al., 2018; Verhaart and Aartsma-Rus, 2019). There will be more clinical trials to be performed in the future, and it will also be applied to other muscle diseases. However, the limitations of AAV are also obvious. Lacking muscle cell tropism induces the inefficiency of AAV. Lacking effective nucleic acid editing targets also makes AAV difficult to use as an effective means to promote ageing skeletal muscle regeneration. Therefore, we propose constructing novel drug delivery systems to repair skeletal muscles with complex pathogenesis.

### Muscle-Targeting Delivery System

Because ageing-related muscle loss has no local lesions, drug therapy can only be administered systemically. The problem with systemic administration is the side effects in other organs, and the high concentration in liver and kidney. To solve above problems, another important strategy for building drug delivery systems is muscle-targeting delivery systems. A muscle-targeting delivery system requires a targeted motif, which can be combined with a delivery system to specifically deliver drugs to skeletal muscles. For example, skeletal muscle-related cell surface-specific proteins can serve as a targeted motif. Large molecules such as antibodies or antibody-drug conjugates would also be a potential method of targeting (Ebner et al., 2015). Several examples of muscle-targeting peptide have been reported. Samoylova TI et al. found a heptapeptide sequence, ASSLNIA, which could improve specificity for binding to skeletal muscle by screening a random phage display library (Samoylova and Smith, 1999). Then, Jativa S D et al. develop a generation 5-polyamidoamine dendrimer (G5-PAMAM) modified with ASSLNIA to synergistically enhance gene delivery to skeletal muscle cells (Jativa et al., 2019). Gao X et al. identified a novel 12-mer peptide (M12) which was shown more effective muscle-homing ability. This motif could enhance binding affinity to myoblast *in vitro*. Conjugated muscle-homing peptide with phosphorodiamidate morpholino oligomers showed significant improvement in grip strength in mdx mice (Seow et al., 2010; Gao et al., 2014). Currently, only a few studies have discovered molecules with muscle-targeting functions. Therefore, subsequent research still needs to explore more novel muscle-targeting molecules and make appropriate modifications to ameliorate ageing-related muscle loss.

### Nanoparticles

The growing concern of nanoparticles is triggered by the rapid development of nanotechnology. Nanoparticles have many unique advantages including enhanced tissue targeting, nucleic acids protection from degradation, and low immunogenicity (Nance et al., 2018; Nag et al., 2020). For example, Poussard, S et al. observed the silica nanoparticles uptake by myoblasts and promote the differentiation *in vitro* (Poussard et al., 2015). Ge. J et al. developed a kind of monodispersed gold and gold-silver nanoparticles (AuNPs and Au-AgNPs) which could support adhesion and proliferation of myoblast by motivating the p38α MAPK signaling pathway (Ge et al., 2018). Raimondo, T.M. et al. found that constructing cytokines binding gold nanoparticles could regulate the inflammatory phase by changing M2 macrophage polarization, which subsequently promote regeneration and increased newly myofibles function (Raimondo and Mooney, 2018). Nanoparticles are easy to prepare on a uniform scale and are easier to modify. There are many optional materials for nanoparticles such as metal particles or silica particles. However, the follow-up research still needs to consider the safety of nanoparticles, including material toxicity, dose toxicity, and metabolic rate. In addition, how to increase the skeletal muscle targeting of nanoparticles still needs to be explored.

### Extracellular Vesicles (EVs)

**Figure 4:**
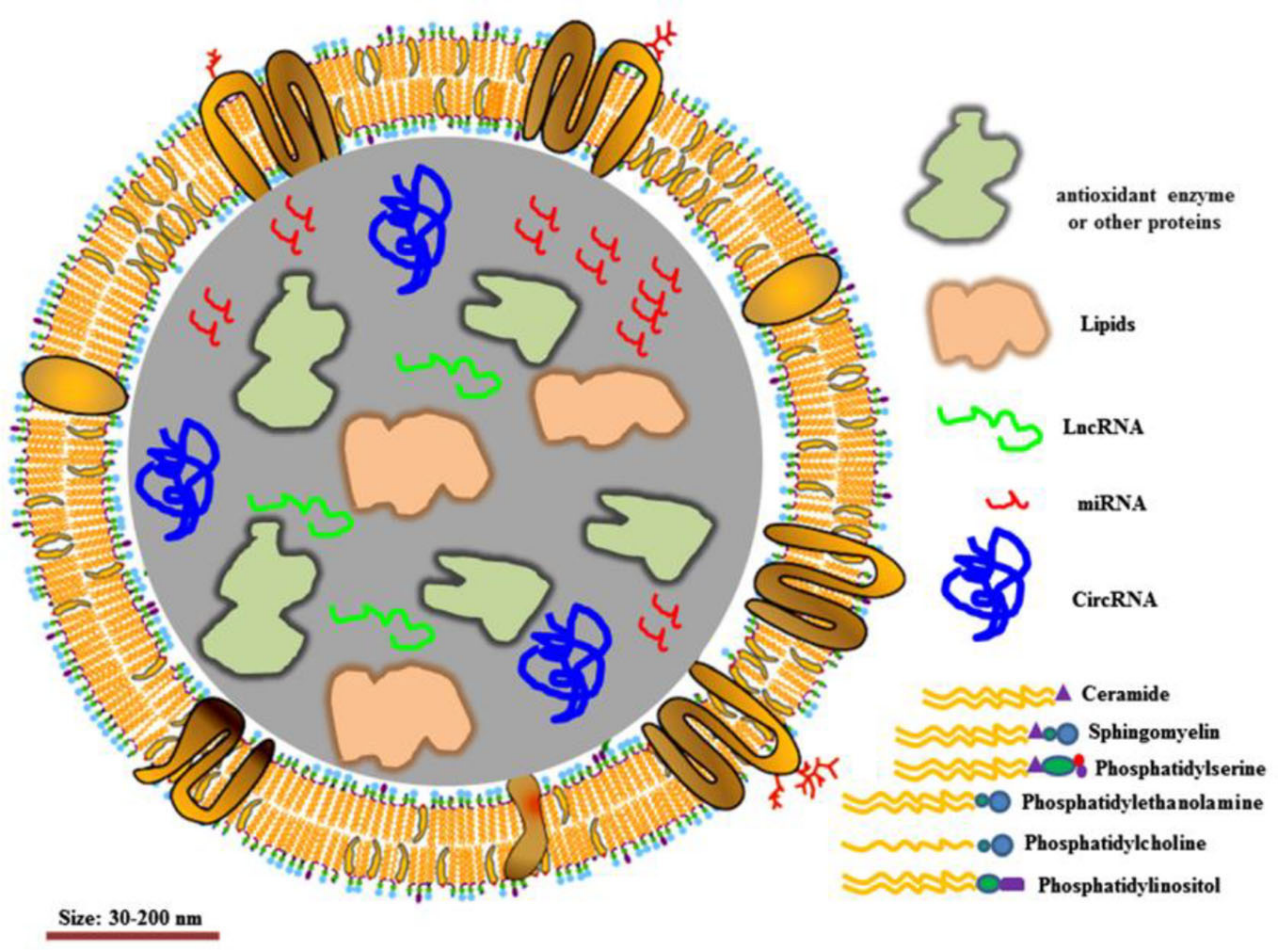
Structure of extracellular vesicles. Reproduced, with permission, from (Rong et al., 2020).

EVs, 30–150 nm in diameter, have been demonstrated to transfer functional proteins, nucleic acid (e.g. mRNA, miRNA, lncRNA) to neighboring or remote cells. The membrane of EVs acts as a natural barrier to prevent degradation in the blood circulation **(** **Figure 4** **)**. EVs can be administered *via* intravenous (IV) and intranasal routes to reach the target location, depending on the therapeutic purpose. Due to these characteristics, EVs are considered ideal natural systems for drug delivery (Vader et al., 2016; Skotland et al., 2020; Rong et al., 2020).

Many studies show that EVs are closely involved in skeletal muscle metabolism and regeneration. Zanotti S et al. found that EVs derived from local fibroblasts in the Duchenne muscular dystrophy (DMD) muscle are able to induce a transfer of unactivated fibroblasts to myofibroblasts thereby motivating the fibrotic response (Zanotti et al., 2018). As mentioned above, Fulzele, S. et al. found EVs-derived miR-34a could regulate muscle metabolism (Fulzele et al., 2019). Many studies investigated that physical exercise, which is closely related with muscle regeneration, can induce rapid release of EVs into the blood circulation. Fruhbeis, C et al. found that a consecutive release of EVs would be triggered after physical exercise. During physical exercise, the released-EVs may participate in intercellular communication or remote communication which involve signaling across tissues and organs (Frühbeis et al., 2015). Guescini, M found similar results and further showed that muscle-specific miRNAs in EVs were significantly upregulated (Guescini et al., 2015). Whitham, M et al. found that exercise-related EVs tend to accumulate in the liver and can transfer inner proteins (Whitham et al., 2018). These studies have demonstrated the promising potential of EVs for therapeutic drug delivery in various muscle diseases. It is worth noting that Ran N et al. created a novel exosome-based delivery system by anchoring the inhibitory domain of myostatin propeptide. The exosome-delivery system could efficiently promote muscle function in mdx mice (Ran et al., 2020). Gao et al. also demonstrated a muscle-targeting exosome-based delivery system, which was modified by CP05, could increase dystrophin expression in mdx mice (Gao et al., 2018). These exosome-based delivery platforms may be effective delivery methods for the treatment of DMD.

The advantages of exosomes are good biological safety, easy absorption and metabolism. Its widespread presence in the body also makes it relatively easy to obtain. It has been found that exosomes have a natural targeting function, which makes it an ideal precision delivery system (Song et al., 2019). However, the research on exosomes is still in an early stage, and the following issues still need to be solved. First, constructing a large number of exosomes with uniform size is still difficult. Efficient loading rate also needs to be considered. In addition, the selection of cell-derived exosomes to construct a delivery system still requires further exploration. At present, there are few studies on the affinity and targeting ability of different exosomes to skeletal muscle. When the problem of exosome preparation is solved, screening the appropriate functional molecule is another key step. In summary, exosomes may be an ideal delivery system for aging skeletal muscle regeneration. Exploring the characteristics and modification of exosomes still needs further investigation.

## Conclusion and Future Directions

Currently there is no ideal treatment for the ageing-related loss of skeletal muscle. Nano-carrier drug delivery system is an emerging area which may provide the ultimate solution for the clinical treatment. Future research should be divided into two aspects for further exploration. Current studies on potential targets for skeletal muscle regeneration do not consider the characteristics of aging. Taking into account the changes in the muscle satellite cells and immune microenvironment in aging skeletal muscle, new therapeutic targets may be discovered. Although the nanocarrier delivery system provides a good method for the treatment of aging diseases, many safety and effectiveness issues still need to be explored. The construction of the delivery system should be carried out simultaneously with the research on the mechanism of regulating muscle regeneration. The ideal delivery system should not only play a role in promoting, but adopt multiple control methods to maximize the improvement of aging muscle loss.

## Author Contributions

PY, LZ, and PT made major contributions to the conception of the work. YL, MC, and YY drafted the manuscript. YZ, ML, YQ, and SC revised the manuscript.

## Conflict of Interest

The authors declare that the research was conducted in the absence of any commercial or financial relationships that could be construed as a potential conflict of interest.
